# Multiple Genome Sequences of *Lactobacillus plantarum* Strains

**DOI:** 10.1128/genomeA.00654-17

**Published:** 2017-07-20

**Authors:** Thomas A. Kafka, Andreas J. Geissler, Rudi F. Vogel

**Affiliations:** Technische Universität München, Lehrstuhl für Technische Mikrobiologie, Freising, Germany

## Abstract

We report here the genome sequences of four *Lactobacillus plantarum* strains which vary in surface hydrophobicity. Bioinformatic analysis, using additional genomes of *Lactobacillus plantarum* strains, revealed a possible correlation between the cell wall teichoic acid-type and cell surface hydrophobicity and provide the basis for consecutive analyses.

## GENOME ANNOUNCEMENT

Cell wall teichoic acids (WTA) are *inter alia* suggested to influence cell adhesion (1, 2). The species *Lactobacillus plantarum* was shown to be unique among this genus to produce either poly(glycerol-3-phosphate) [poly(Gro-P)] or poly(ribitol-3-phosphate) [poly(Rbo-P)] WTA molecules, depending on the strain’s gene equipment, possibly resulting in different alditol-polymer-dependent cell surface characteristics (3–6). Testing the surface hydrophobicity of different *L. plantarum* strains by the MATH test, large differences in surface hydrophobicity could be determined (T. A. Kafka, D. Reitermayer, C. A. Lenz, and R. F. Vogel, unpublished data). In order to gain insights into the role of WTA type on cell surface hydrophobicity, we sequenced the complete genomes of four strains that vary in cell surface hydrophobicity.

Surface hydrophobicity was determined by a modified version of the MATH test (7). High-molecular-weight DNA was purified from de Man-Rogosa-Sharpe (MRS) liquid cultures using the Genomic-tip 100/G kit (Qiagen, Hilden, Germany). Using NanoDrop (Thermo Fisher Scientific) and agarose gel electrophoresis, the quality and quantity of isolated genomic DNA were checked. Single-molecule real-time sequencing (PacBio RSII) was carried out at GATC Biotech (Constance, Germany) (8). An insert size of 8 to 12 kb was selected for library creation, resulting in at least 200 Mb of raw data from 1 to 2 SMRT cells (1 × 120-min movies), applying P4-C2 chemistry. Assembly was done with SMRT Analysis version 2.2.0.p2, using the Hierarchical Genome Assembly Process (HGAP) (9), and completed by manual curation (https://github.com/PacificBiosciences/Bioinformatics-Training/wiki/Finishing-Bacterial-Genomes). Genomes were annotated using the NCBI Prokaryotic Genome Annotation Pipeline (PGAP) (10).

Strain characteristics, sequencing statistics, genome information, and accession numbers are listed in Table 1.

**TABLE 1:** Strain characteristics, sequencing statistics, genome information, and accession numbers

Strain	Source	Surface hydrophobicity^a^	WTA type	BioSample no.^b^	Accession no.^c^	Coverage (×)^d^	Size (Mb)	No. of contigs^e^	G+C content (%)	No. of CDSs^f^
TMW 1.708	Raw sausage	Highly hydrophilic	Poly(Gro-P)	SAMN05805046	CP017374–CP017378	250	3.24	5	44.5	2,815
TMW 1.25	Raw sausage	Highly hydrophobic	Poly(Rbo-P)	SAMN05805044	CP017354–CP017362	290	3.35	9	44.3	2,944
TMW 1.277	Palm wine	Highly hydrophobic	Poly(Rbo-P)	SAMN05805045	CP017363–CP017373	247	3.40	11	44.2	2,987
TMW 1.1623	Unknown	Moderately hydrophobic	Poly(Rbo-P)	SAMN05805047	CP017379–CP017383	237	3.33	5	44.3	2,919

a Determined for stationary-phase cells using a modified version of the MATH test (7).

b All BioSamples are part of BioProject PRJNA343197.

c Accession numbers are listed for all contigs of each whole genome (as a range).

d Average coverage of HGAP assembly.

e In chromosome plus plasmids and partial plasmids.

f CDSs, coding sequences (total) based on NCBI PGAP.

The chromosome sizes range from 3.09 Mb to 3.14 Mb, with G+C contents of 44.6% to 44.7%. We found four to 10 plasmids (per strain), with G+C contents ranging from 35.0% to 55.0%. Plasmid sizes range from 0.8 to 67.9 kb, resulting in genome sizes of 3.24 to 3.40 Mb. The chromosomes encode 64 to 69 tRNAs.

The analysis of all four *L. plantarum* genomes, considering additional genomes of already sequenced *L. plantarum* strains, revealed conserved differences in WTA biosynthesis clusters, resulting in two different WTA types possibly correlating with specific surface hydrophobicities. In hydrophobic and hydrophilic stains, we could determine the *tar* locus, which is necessary for the biosynthesis of poly(Rbo-P) WTAs (3). Thereby, we could prove that the *tar* loci of hydrophilic and hydrophobic strains differ by sharing gene sequence identities of only 65 to 87% and that these differences are conserved among these two groups (99% sequence similarity, 99% coverage to each other). Comparing the genomes of both groups by BADGE and following bioinformatic analysis, we could determine the genes *tagD1-tagF1-tagF2* (*tag* locus) in hydrophilic strains, which were lacking in the genomes of hydrophobic strains (11). In line with that finding, hydrophilic strains are supposed to synthesize poly(Gro-P) while hydrophobic strains are supposed to synthesize poly(Rbo-P) WTAs (3, 4).

The availability of these *L. plantarum* genome sequences provides the basis for consecutive analyses (e.g., wall teichoic acid isolation and transcriptomics) with the objective to obtain new insights regarding the role of WTAs on surface hydrophobicity or adhesive properties to biotic and abiotic materials.

### Accession number(s).

The four complete *L. plantarum* genomes have been deposited in DDBJ/EMBL/GenBank under the accession numbers stated in Table 1.
